# Memory persistence: from fundamental mechanisms to translational opportunities

**DOI:** 10.1038/s41398-024-02808-z

**Published:** 2024-02-14

**Authors:** Santiago Abel Merlo, Mariano Andrés Belluscio, Maria Eugenia Pedreira, Emiliano Merlo

**Affiliations:** 1 Instituto de Fisiología, Biología Molecular y Neurociencias, Consejo Nacional de Investigaciones Científicas y Técnicas and Universidad de Buenos Aires, Buenos Aires, Argentina; 2 Instituto de Fisiología y Biofísica Bernardo Houssay, Consejo Nacional de Investigaciones Científicas y Técnicas and Universidad de Buenos Aires, Buenos Aires, Argentina; 3 Laboratorio Bases Neuronales del Comportamiento, Departamento de Ciencias Fisiológicas, Facultad de Ciencias Médicas, Universidad de Buenos Aires, Buenos Aires, Argentina; 4 School of Psychology, University of Sussex, Falmer, UK

**Keywords:** Long-term memory, Addiction

## Abstract

Memory persistence is a double edge sword. Persistence of adaptive memories is essential for survival and even determines who we are. Neurodegenerative conditions with significant memory loss such as Alzheimer’s disease, testify how defects of memory persistence have severe and irreversible effects on personality, among other symptoms. Yet, maintenance of overly strong maladaptive memories underlies highly debilitating psychiatric conditions including post-traumatic stress disorder, specific phobia, substance dependence and binge eating disorder. Here we review the neurobiological mechanisms supporting memory formation, persistence, inhibition and forgetting. We then shift the focus to how such mechanisms have been exploited to alter the persistence of laboratory-generated memories in human healthy volunteers as a proof of concept. Finally, we review the effect of behavioural and pharmacological interventions in anxiety and addiction disorder patients, highlighting key findings, gaps, and future directions for basic and translational research.

Memory is a fundamental attribute of animal cognition that allows individuals to store and retrieve information about past experiences. The interplay between the processes of maintenance and inhibition enables memory to fulfill its primary role, to use previous experiences to optimise decision-making and fitness in a constantly evolving and unpredictable environment [1]. Current hypotheses state that memories are stored through maintenance of synaptic connections between neuronal subgroups and are not immutable. They can be challenged by active forgetting, and retrieval-dependent mechanisms triggered by memory prediction error; as well as subject to decay via inhibitory processes that limit memory persistence. The balance between maintenance and inhibition mechanisms will ultimately determine memory existence, modulating its strength and duration. Here we review the neurobiological mechanism of memory formation, persistence and forgetting with evidence from human and non human animals.

## Mechanisms of memory formation

There are multiple forms of memories, serving non-overlapping biological functions, which can be classified according to their duration and information content (for a review see [2]). Long-term memories refer to learned past experiences that can be recalled when these are no longer present in the stream of thought, days, months, or decades after they have been acquired.

Long-term memories can be further divided into nondeclarative and declarative [3]. The first are dispositional memories (e.g., associative, and nonassociative), that are expressed by behavioural changes rather than recollection, such as procedural memory that consists of storing the motor and executive skills necessary to perform a specific action. Declarative memories (i.e., episodic, and semantic) are representational and provide a model of the external world that can be recollected and guide behaviour [3].

### Memory formation: neuronal and molecular mechanisms

**Fig. 1:**
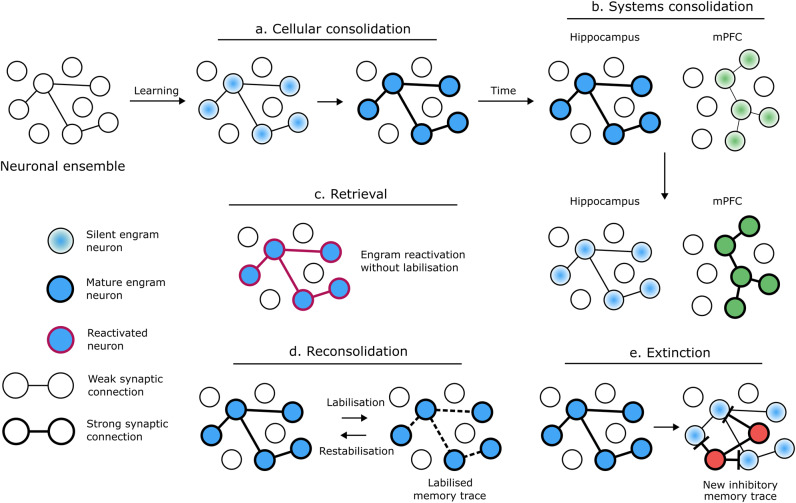
The fate of the memory engram through different memory processes. **a** After learning, the memory engram is stabilised through cellular consolidation and forms a long-term memory with mature neurons and strong synaptic connections. **b** With the passage of time, long-term memories undergo a circuitry reorganisation through systems consolidation where the hippocampus and the medial prefrontal cortex are key for this process. **c** Memory retrieval requires reactivation of the neuronal ensemble formed during learning, without trace labilisation. **d** With reconsolidation, memory returns to a labile state during a certain time window, opening the possibility of memory disruption and modification, followed by a restabilisation process. **e** Extinction does not erase the original memory but promotes the formation of a new inhibitory memory trace controlling behavioural output.

At the neuronal level, remembering involves the retrieval of a past brain state into a present brain state through the activation of specific neurons, which serve as the basis for memory engrams [1]. Memories are thought to be codified in sparsely distributed neuronal groups or ‘assemblies’ arising from the strengthening of synaptic contacts between neurons co-activated during learning [4]. Thanks to recent technological advances, it is now possible to characterise the nature and persistence of memory ‘assemblies’ or ensembles (e.g., TetTag system [5]) through tagging and manipulation of neurons activated during learning. The manipulation studies aim to elucidate whether these neuronal ensembles are ‘necessary’ or ‘sufficient’ for the target memory. Typically, to demonstrate necessity of ensemble function, researchers inhibit neuronal ensemble activity and observe its effects on memory expression [6]. By inhibiting these neuronal groups, it is possible to test whether memory retrieval requires reactivation of the neuronal ensemble formed during learning (Fig. 1c). Also, these techniques allow testing for sufficiency. Activation of the same neuronal pattern observed at encoding results in memory expression, indexed by a specific behavioural manifestation [7].

The establishment of memory neuronal ensembles requires changes in the connectivity between participating neurons by a process called synaptic plasticity, which involves the adjustment of synaptic connectivity through long-term potentiation (LTP) and long-term depression (LTD) [8]. LTP leads to an increase in synaptic transmission between neurons, while LTD results in a decrease in synaptic strength. Studies have demonstrated a strong correlation between memory formation and LTP, and memory inhibition and LTD (for a review see [9, 10]).

At the molecular level, long term memories are formed by a process called cellular consolidation, which induces long lasting changes in synaptic contacts between memory ensemble neurons (Fig. 1a; for a review see [11]). Upon synaptic activation during a memorable experience, a series of molecular events lead to expression of specific genes and protein synthesis, which promote and maintain long-term changes to synaptic connectivity. Amongst the myriad of molecular events engaged during memory consolidation, protein kinases and phosphatases (K&P) are key players [12].

The balance between K&P activity not only determines whether a memory can be formed, but also its strength. Among the various kinases and phosphatases involved in memory formation, the recruitment of the mitogen-activated protein kinase/extracellular signal-regulated kinase (MAPK/ERK) and Ca^2+^- and calmodulin-dependent protein phosphatase calcineurin (CaN) offer an illustrative example. In rodents, pharmacological inhibition of MAPK/ERK prevents the consolidation of an aversive associative memory [13]. On the contrary, CaN inhibition during memory acquisition increases the synthesis of the transcription factor Zif268 and strengthens conditioned taste aversion, making it more resistant to memory extinction [14]. However, while transgenically overexpressing Zif268 strengthens conditioned taste aversion [14], conflicting findings have demonstrated that this effect depends on Zif268 dosage [15]. In heterozygous Zif268^+/−^ mice, carrying half the complement of the protein, strengthening of contextual fear memory is observable, but blocked in homozygous Zif268^-/-^ mice [16].

### Remote memories and systems consolidation

To be maintained for months and beyond, long-term memories undergo a circuitry reorganisation through systems consolidation (Fig. 1b) [17]. As opposed to cellular consolidation, systems consolidation refers to memory maturation and reorganisation in the underlying neural substrate.

Hippocampal lesions in humans result in anterograde amnesia for episodic memories and retrograde amnesia for memories acquired shortly before the lesion, but leave older memories intact [18]. The hippocampus is important for the formation and early retrieval of memories, by forming an index of the cortical structures that were active during learning and are responsible for storing the memory content [19]. Weeks or months after acquisition, memories can be retrieved independently of the hippocampus, suggesting the memory trace has been reorganised [20]. Moreover, cortical neurons essential for remote memory retrieval are recruited during learning but remain silent until systems consolidation has taken place [21]. During learning, medial prefrontal cortex (mPFC) tagged neurons form a silent memory engram, retrievable by optogenetic activation but not natural recall cues [22]. Through systems consolidation, the neural signature required for retrieval migrates from the hippocampus to the neocortex through the maturation of these silent engrams in the mPFC, a process dependent on hippocampal inputs as seen in circuitry and oscillatory activity studies [23]. The last step in systems consolidation involves de-maturation and silencing of the hippocampal engram, possibly through reorganisation of synaptic connections based on erasure of old connections and creation of new ones through new-born neurons [24]. The role of the mPFC in remote episodic memories may be equivalent to that of the hippocampus for recent memories [20], but additionally integrating individual components of engrams stored in various other cortical areas [25].

## Memory decay through forgetting

### Retrieval induced forgetting in humans and other animals

The selective suppression of outdated memories is an actively promoted phenomenon in the brain. This process enables behavioural adaptability in changing environments by discarding information that could interfere with adaptive behavioural control. Humans selectively retrieve relevant information in part by inhibiting competing traces through retrieval-induced forgetting (RIF) [26]. This mechanism can shape ongoing memory accessibility based on behavioural, cognitive, and emotional requirements [27]. The RIF phenomenon has been extensively studied in humans using the retrieval-practice paradigm developed by Anderson et al. [26] In this paradigm, individuals study a series of category-exemplar pairs (e.g., fruit-banana, metal-gold, fruit-apple) and then retrieve only half of the items from half of the categories when provided with specific cues at retrieval (e.g., fruit-ba___). In his original paper, Anderson et al. found that practised exemplars (Rp +) were better remembered in a subsequent test session than non-practiced exemplars from practised categories (Rp-). At the same time, Rp- items were less well remembered as items from non-practiced categories (Nrp), indicating that remembering some items from a category caused active forgetting of the rest of the items from the same category. It is hypothesised that the mechanism behind RIF is active memory inhibition [28]. Trying to remember a specific item following cue presentation, causes multiple items to be activated and generates competition between them. Thus, inhibition mechanisms are activated to reduce interference from non-target items during retrieval of target ones. However, some RIF may be due to retrieval disruption. Recalling a subset of words from a list would disrupt the original organisation of the list in memory, making the non-practiced words less accessible at retrieval [29]. Hence, retrieval induced forgetting may be a combination of effects arising from active memory inhibition and disruption of retrieval cues triggered by partial practice sessions.

The process of active forgetting is also present in non-human animals. Bekinschtein et al. [30] described an animal model of RIF in which the prefrontal cortex (PFC) suppresses competing memories and initiates a signal that triggers active forgetting. In this study, rats were trained in a novel object recognition paradigm to associate an environment with two objects (A and B). After this, memory for object A was repeatedly retrieved by exposing the animal to object A in the same context, along with a novel object (C) each time. Repeatedly retrieving the memory of A impaired subsequent retrieval of the competing memory of B. Thus, remembering caused forgetting. At the neural level, RIF was dependent on PFC activation, as injections of the GABA-A receptor agonist muscimol in this region suppressed the effect. This effect in rats is consistent with previous studies in humans, which showed that impairing PFC function selectively abolishes RIF [31].

### Memory forgetting molecular substrates

Forgetting may be mediated by disassembling the neuronal ensembles encoding past experiences. Passive memory forgetting is a consequence of biological dismantling of memories due to molecular turnover. When this process is actively promoted, it has a direct effect in synaptic connectivity [32].

Both learning and memory, as well as the expression of long-term potentiation (LTP), increase the availability of GluA2 containing α-amino-3-hydroxy-5-methyl-4-isoxazolepropionic acid type glutamate receptors (AMPARs) in the postsynaptic membrane. Consequently, forgetting involves removal of AMPARs from the postsynaptic sites through receptor internalisation [33]. The internalisation signal includes calcium influx via N-methyl-D-aspartate type glutamate receptor (NMDAR) activation. NMDARs are heterotetramers made up of two obligatory GluN1 subunits, and GluN2 or GluN3 subunits [34]. Expression of alternative GluN2 variants (i.e., A, B, C, and D) vary in different brain regions along development [35], but the balance between GluN2A and GluN2B expression has direct effects on synaptic strength and memory persistence. GluN2B subunit containing NMDARs favours calcium entry [36] and have a bigger impact on synaptic strength by direct modulation of AMPARs expression in the synaptic membrane [37]. Thus, NMDAR activity is key for active memory decay where greater activation accelerates long-term memory loss, while lower activation slows it down [37]. This hypothesis is supported by the fact that blocking NMDAR activity in the dorsal hippocampus prevents the natural forgetting of object location in an open field arena [38] and spatial memories in rats [39].

Memory strength and age are also associated with differential involvement of NMDARs subtypes. Learning experiences that form stronger long-term memories, such as those that contain emotional significance, are associated with lower expression of GluN2B-NMDAR in the amygdala [40]. Also, recently formed memories are associated with lower GluN2B-NMDAR levels compared to older memories.

In conclusion, the balance in the expression of GluN2A- and GluN2B-containing NMDARs regulates active memory forgetting and defines memory destiny by affecting the rate at which AMPARs are removed from post-synaptic sites.

## Memory potentiation and inhibition after retrieval

Fully consolidated associative memories, formed by learning a predictive association between an emotionally irrelevant environmental stimulus (conditioned stimulus, CS) and a biologically relevant outcome (unconditioned stimulus, US), are not immutable entities but can be altered following memory retrieval. Updating associative memories upon novel information is an evolutionarily conserved feature that maximises animal welfare and survival. Once a CS-US memory is fully consolidated, it can be retrieved by partially or fully re-experiencing the learned experience, evoking the brain state that was active during learning. If memory retrieval induced by cue (CS) exposure takes place in presence of new or unexpected information (i.e., the CS is no longer contingent with the US) a prediction error signal is generated, which modulates memory persistence [41, 42].

### Memory potentiation or inhibition depends on the extent of prediction error

Under certain retrieval conditions, memories can be either potentiated or inhibited by prediction error (PE), defined as a mismatch between what is expected (i.e., the CS is followed by the US) and the actual event (i.e., CS alone) [43]. Retrieval can be understood as a cue-induced ‘reawakening’ and behavioural expression of the memory engram [44]. If PE is moderate, the memory undergoes a so-called reconsolidation process (Fig. 1d), by which it returns to a time-dependent labile state, allowing for memory disruption or modification, followed by a restabilisation process [45]. Within the lability window, memories may be disrupted by protein synthesis inhibitors [46, 47], β-adrenergic [48, 49], or glucocorticoid receptor antagonists [50]. Importantly, memories can be updated in strength and content during reconsolidation [51–53]. Although PE is a necessary condition for memory reconsolidation, it is not sufficient [43, 54]. Larger PE signals, produced by repeated or prolonged presentation of the CS alone, leads to inhibition of the original memory by extinction (Fig. 1e). Since extinction is not permanent, with the original memory eventually returning, it does not erase the CS-US memory but promotes the formation of a new inhibitory memory trace (CS-noUS) that competes for behavioural control [55]. Memory age and strength modulate the relationship between PE, memory maintenance and inhibition, with older and stronger memories requiring longer PE signals to undergo memory reconsolidation or extinction [46, 56] (for a review see [15]).

### Preventing memory return

Consistent with the idea of PE as the switch between memory updating and inhibition, Woods and Bouton [57] showed that occasional reinforcements during response elimination can reduce the rate of reacquisition in an operant lever-pressing task compared to traditional extinction procedure. This effect could be due to an overall reduction in PE through sporadic repetition of acquisition conditions by occasional reinforcements. Other procedures have been developed to enhance extinction and to slow the return of the original memory. One of such procedures is counterconditioning, by which behaviour is modified through learning of a new association with an outcome of opposite valence to the original outcome [58]. Counterconditioning is considered to rely on integration of an opposite valence information during retrieval into the background of the original trace, reducing its outcome. Another procedure that was effective in potentiating the effects of extinction is that of retrieval-extinction. When an extinction procedure (i.e., repeated CS alone presentations) is applied shortly after memory reactivation, during the reconsolidation window, it delays or permanently suppress spontaneous or stimulus-induced recovery of the original memory [59, 60]. This post-consolidation behavioural manipulation may open the original memory for updating or rewriting, which may explain the lack of recovery from extinction [60] (but also see [61]).

Recent models have proposed that a retrieval session resulting in large PE serves as a segmentation signal, indicating a novel state is responsible for the experienced facts and promoting new associations [62]. When conditions change from reinforced trials during conditioning to non-reinforced trials in extinction learning, the animal infer the existence of a different latent cause than the one that was active during conditioning, driving the formation of a new inhibitory trace. This can explain why the traditional extinction procedure leads to the formation of a new memory trace [63], allowing the original memory to persist unmodified and express under certain conditions [64]. Under this Bayesian learning framework called latent cause model, the subject learns about associations between “causes” and “outcomes” as a form of clustering whereby observations are clustered together according to their hypothetical latent causes. Using these ideas, Gershman et al. have developed a new strategy to prevent the return of the original association called gradual extinction. It consists of reducing prediction error enough to prevent the formation of a new memory, and this was achieved by gradually reducing the frequency of aversive stimuli, rather than eliminating it abruptly, which prevents the return of fear by canonical manipulations [64].

## Memories in humans, from bench to bedside

### Affecting memory persistence in healthy subjects

In humans, memories can become maladaptive, causing distress, and impairing daily functioning [65]. Research from preclinical models have inspired a myriad of studies targeting declarative and nondeclarative memories in humans, aiming to maintain the good ones and suppress the bad ones. In this section we will review studies involving pharmacological or behavioural interventions aimed at altering memory persistence in healthy volunteers.

The beta-adrenergic receptor antagonist propranolol, which is commonly used to treat hypertension and anxiety disorders, can affect fear memories. Participants that underwent fear conditioning treated with propranolol in conjunction with memory reactivation, exhibited reduced fear response compared to matched placebo controls [49]. Moreover, propranolol can disrupt fear even if the memory was reactivated by exposure to the US alone, resulting in fear response reduction to multiple CSs previously associated with that US [66]. However, some recent studies have failed to replicate the effects of propranolol on fear memory reconsolidation due to a failure to engage memory destabilisation and have questioned whether treatments based on reconsolidation blockade are robust enough for clinical translation [67]. Another compound tested for treating maladaptive memories is D-cycloserine (DCS). DCS is an N-methyl-D-aspartate (NMDA)-type glutamate receptor partial agonist and can enhance learning and memory processes [68]. In laboratory animals, DCS systemic or intra-basolateral amygdala administration facilitates extinction of conditioned fear in rats, presumably promoting memory consolidation [69, 70] (for a review see [71]). Notably, memory extinction facilitation only occurs when there is within-session fear reduction [72]. Randomised, blind, placebo-controlled trials in healthy participants show that fear memory extinction is augmented by prior administration of DCS [73]. This evidence indicates that persistence of lab generated fear memories in healthy human volunteers can be altered by relatively simple pharmacological interventions applied at retrieval. Hence, reducing fear by blocking memory reconsolidation or enhancing memory extinction is a promising avenue for improving current treatments for anxiety disorders such as post-traumatic stress disorder (PTSD) or specific phobias.

Behavioural interventions aimed at diminishing the persistence of aversive memories have also been tested in recent years. Research has shown that working memory, particularly the visual-spatial component, is involved in the formation and retrieval of traumatic memories [74]. Holmes et al. [75] reported that playing the videogame “Tetris” during memory encoding may interfere with traumatic memory formation by occupying the visual-spatial working memory system. Participants who played Tetris after watching a traumatic film reported significantly fewer flashbacks and intrusive memories compared to those in the control group [75]. Moreover, if participants played Tetris shortly after retrieval of an experimental traumatic memory, subsequent emotional memory intrusions are also reduced compared with controls that only retrieved the memory [76].

The so-called retrieval-extinction manipulation is another behavioural intervention to reduce aversive memory persistence. Monfils et al. [60], among others, have shown that in rats, fear memory retrieval followed shortly after by an extinction session produced longer-lasting fear reduction than extinction-only treatment. Nevertheless, based on a meta-analysis, Kredlow and colleagues demonstrate the moderate effect of such treatment for aversive memories [77]. As in animal models, the effect of such manipulation to reduce fear memory persistence in humans has been controversial [78, 79]. Discrepancies between these studies may be partly due to large individual differences, inconsistent engagement of memory reactivation in all participants, and the experimental limitation of not having a reliable human biomarker indexing memory destabilisation [42].

Strengthening episodic and non-episodic memories may be key for the treatment of Alzheimer’s or other disorders characterised by memory loss. In laboratory animals, strengthening memories by further training requires memory reconsolidation [80], suggesting this process may prevent natural memory decay. In humans, reactivation of fully consolidated memories for declarative memories makes them last longer, maintain precision, and resist interference [52, 81]. Pharmacological interventions can also promote memory persistence. In healthy human volunteers, DCS enhances declarative and discriminatory learning when administered during acquisition [82] or before sleep [83], an effect probably mediated by enhanced hippocampal activity [82].

This evidence strongly supports pharmacological or behavioural manipulations targeting well-established memory processes that can be exploited to alter the persistence of laboratory-generated memories in healthy volunteers. In the following sections we will review studies using such tools to alter memories in patients suffering from maladaptive memories.

### Preventing memory loss

Memory impairments are frequently observed as a prevalent symptom in various neurological and psychiatric disorders. For individuals dealing with these conditions, the loss of critical memories can have profound consequences on their daily life, relationships, and overall well-being. Preserving essential target memories could play a pivotal role in maintaining a good quality of life for patients. Research and advancements in the field of neuroscience and psychology are continuously seeking ways to improve memory retention and support those affected by memory impairments. Novel approaches such as cognitive training, neurofeedback, and virtual reality (VR) are emerging as potential therapeutic strategies for individuals with memory impairments. Cognitive training involves practising specific cognitive tasks with the goal of improving cognitive function. In patients with idiopathic Parkinson disease, cognitive training has shown to improve new episodic learning as well as memory retention [84]. Neurofeedback is a technique that involves training individuals to regulate their own brain activity using real-time feedback from brain activity recordings from EEG or fMRI. Neurofeedback has shown promise as a potential therapeutic approach for individuals with memory impairments. Both healthy individuals and those with traumatic brain injury showed improved memory performance with neurofeedback [85]. VR can be used to create immersive environments that can simulate real-world situations and enhance memory performance by providing multisensory experiences. This technology has shown promise as a potential therapeutic approach. VR-based memory training can improve memory performance in older adults with mild cognitive impairment [86]. These technologies are emerging as promising approaches to maintain memory precision and offer potential therapeutic applications for improving cognitive function and quality of life in individuals with memory impairments.

Pre-clinical Alzheimer’s disease patients show defects on the highly demanding face-name associative memory [87]. Reconsolidation-based interventions can improve face-name pair memories making them longer lasting and with less errors/omissions. Targeting face-name pair memory at reactivation improves memory retention in young and old healthy volunteers, but also in mild cognitive impaired subjects, suggesting that inducing memory reconsolidation can boost memory persistence [88] even in patients suffering from memory loss.

### Disrupting maladaptive memories

The formation and persistence of maladaptive memories are significant contributors to the development and maintenance of a variety of mental health disorders, including reward-seeking behaviours, depression, and anxiety disorders. As mentioned above in the healthy subjects’ section, retrieval-dependent memory interventions have emerged as a promising approach to reducing or removing maladaptive memories [89].

Anxiety disorders are one of the most prevalent mental health conditions, affecting millions of individuals worldwide, and significantly impairing daily functioning and quality of life [90]. Traditional treatments for anxiety disorders, such as cognitive-behavioural therapy, are effective for many patients, but some individuals do not show benefits, and others experience relapse shortly after treatment. In recent years, research has focused on augmenting retrieval dependent memory manipulations effects through pharmacological interventions to weaken behavioural manifestations of traumatic memories. A paradigmatic example of these novel interventions is propranolol. Administering the beta-adrenergic receptor antagonist upon spider memory reactivation in subclinical spider phobic participants reduced fear to spiders for at least one year [91]. However, this intervention showed contrasting results when used to target spider phobia in clinical participants, where placebo-treated participants showed as much benefit as those receiving propranolol [92]. In PTSD patients, propranolol in conjunction with script-driven traumatic memory reactivation reduced physiological responses to trauma-related cues and, more importantly, PTSD symptoms for at least 4 months [93]. More recent analysis of a bigger set of pharmacological studies in PTSD patients shows more heterogeneous outcomes [94], indicating more research is needed in this potentially groundbreaking area.

Behavioural interventions such as the Reconsolidation of Traumatic Memories (RTM) have also shown promising effects in ameliorating PTSD symptoms in male veterans with intrusive flashbacks and nightmares [95–97]. RTM is based on updating the traumatic memory after its reactivation by weakening the pathological affective response. After treatment, the event becomes available to declarative memory without evoking the strong pathological emotion characteristic of PTSD. Also, within the behavioural interventions, playing Tetris after a memory reminder cue reduced traumatic memory intrusions in trauma-exposed patients [98] and intensive care unit staff [99], two sub-populations with increased vulnerability to disease onset [100].

Reward memory disruption via pharmacological or behavioural interventions has been a topic of increasing interest in the field of addiction research [101]. Systemic administration of ketamine, an NMDAR antagonist, shortly after a brief retrieval of maladaptive cue-alcohol memories reduced alcohol consumption in harmful drinkers for up to 9 months [102]. This effect was more pronounced in participants treated with ketamine after experiencing prediction error at retrieval, compared with ketamine alone, indicating that the restabilisation of cue-alcohol memories was disrupted by the intervention. In another group of hazardous drinkers, inhalation of the NMDAR antagonist gas nitrous oxide (N_2_O), reduced alcohol-seeking if retrieval produced prediction error compared with no surprise conditions [103]. Behavioural manipulations also reduced alcohol consumption in problematic drinkers. Counterconditioning, consisting of presenting beer images preceding consumption of a highly bitter solution, administered after prediction error inducing retrieval also reduced total alcohol consumption in hazardous drinkers for up to 9 months [104]. However, this strategy was not effective for all types of reward memories. Memantine did not reduce smoking behaviour or smoking-related cue reactivity [105], suggesting limited translational feasibility of memory reconsolidation blockade for smoking cessation. Propranolol in combination with reward memory reactivation also showed limited success in reducing drug craving [106]. The retrieval-extinction intervention applied to detoxified heroin addicts showed significant attenuation in heroin craving that persisted for at least six months [107]. In smokers, retrieval-extinction reduced craving for smoke-related cues and cigarette consumption for at least one month post-treatment [108]. Finally, a systematic review and meta-analysis of clinical and sub-clinical populations revealed that memory reactivation-based interventions can reduce or even eliminate maladaptive reward seeking behaviour [109], noting that intervention timing, emotionality and nature of target memory influence their efficacy.

Although this evidence is encouraging, more studies are needed to determine whether naturalistic reward and traumatic memories are malleable by reconsolidation-based interventions. Also, potential off-target memory effects, such as weakening of non-pathological memories, needs to be carefully established before such treatments become a reality.

### Suppressing maladaptive memories by extinction enhancement

Memory extinction-based interventions (e.g., cognitive behavioural therapy, CBT) are well-established to treat anxiety disorders, but they only benefit around 50% of patients [90]. Thus, enhancing memory extinction through pharmacological and behavioural interventions may improve treatment efficacy.

The main pharmacological intervention tested for such purpose is the systemic administration of D-cycloserine [110]. DCS augmented CBT effects for social anxiety disorder but showed mixed results for PTSD patients [111]. Notably, DCS efficacy in enhancing traumatic memory extinction depends on the retrieval dependent memory process engaged during the CBT sessions. At an individual level, if CBT results in memory reconsolidation, DCS will worsen treatment outcome presumably by promoting traumatic memory persistence. Otherwise, if CBT results in effective memory extinction (i.e., within session behavioural change) DCS improves symptom reduction. Given that DCS should be administered before cue exposure to promote extinction, and that treatment outcome relies on effective within session extinction, future attempts to use the drug as a cognitive enhancer for anxiety disorder treatment should ensure an online behavioural monitoring is in place. If cue exposure fails to achieve behavioural extinction, DCS may worsen PTSD symptoms and resistance to further treatment.

In addiction disorders, the role of DCS in promoting maladaptive memory extinction may be more limited. In double-blind, placebo-controlled trial with non-treatment-seeking heavy smokers, DCS oral administration had no effect in nicotine craving or dosing [112]. A similar lack of DCS effect was observed when treating heavy drinkers [113].

In addition to pharmacological interventions, several behavioural techniques have been proposed to enhance memory extinction. One such technique is the use of virtual reality exposure therapy (VRET), which allows individuals to gradually confront feared stimuli in a controlled and safe environment. VRET is as effective as traditional exposure therapy and may be particularly useful for individuals who have difficulty accessing or engaging in in vivo exposure [114]. Other behavioural techniques that have been proposed to enhance fear extinction include cognitive restructuring [115], attentional bias modification [116], and mindfulness-based interventions [117]. These techniques aim to modify the way individuals perceive and respond to feared stimuli, leading to more adaptive and less anxiety-provoking reactions.

In conclusion, there are several pharmacological and behavioural interventions that have been proposed to enhance fear extinction. While some interventions have shown promising results, further research is needed to fully understand their efficacy and optimal implementation.

### Closing remarks

Since Hebb’s proposal in the mid twentieth century that memories are codified by neuronal ensembles, we have established key neurobiological mechanisms responsible for memory formation, maintenance, and forgetting. Even though there is a long way to go in understanding these processes, we can leverage current knowledge to alter memory persistence in health and disease. We have presented here the most promising avenues to promote persistence of adaptive memories, as well as suppression of maladaptive ones in both human and non human animals. Learning more about memory neurobiology and intervention effects will be key to develop novel treatments for some of the most prevalent and debilitating psychiatric conditions.
